# Timing of Kirschner wire removal in pediatric supracondylar humerus fractures: Is early removal a safe and effective option?

**DOI:** 10.55730/1300-0144.6207

**Published:** 2026-03-12

**Authors:** Mustafa Kemal YENİDÜNYA, Sinan YÜKSEL, İbrahim Faruk ADIGÜZEL, Serhat DİNÇ, Önder ERSAN

**Affiliations:** Department of Orthopaedics and Traumatology, Faculty of Medicine, University of Health Sciences, Etlik City Hospital, Ankara, Turkiye

**Keywords:** Supracondylar humerus fracture, Kirschner wire, pin removal timing, pediatric orthopedics, radiation exposure

## Abstract

**Background/aim:**

The optimal timing of Kirschner wire removal following the surgical treatment of pediatric supracondylar humerus fractures remains controversial. This study aimed to compare the clinical and radiographic outcomes of early versus late K-wire removal and to contribute to the standardization of the timing of pin removal in pediatric patients.

**Materials and methods:**

This retrospective study included 90 pediatric patients (aged 4–14 years) treated with closed reduction and percutaneous pinning for Gartland type III and IV supracondylar humerus fractures. Patients were grouped according to the timing of pin removal as early (postoperative days 21–28, n = 40) or late (after postoperative day 28, n = 50). Clinical outcomes were evaluated in the early postoperative period and at 6 months using the Mayo Elbow Score and Flynn criteria, whereas radiographic assessments at 6 months included the Baumann angle and lateral rotation percentage. Age-based subgroup analyses were performed for patients aged ≤8 years and >8 years.

**Results:**

At 6 months, the Baumann angle did not differ between groups, whereas lateral rotation percentage was significantly lower in the early removal group (p = 0.03). Overall clinical outcomes were similar between the early and late removal groups. In subgroup analyses, patients aged ≤8 years showed significantly better early postoperative Mayo Elbow Score and Flynn criteria results after early pin removal (p < 0.05), whereas no differences were observed in older patients. Clinical outcomes at 6 months were comparable across all groups.

**Conclusion:**

Early and late K-wire removal result in comparable midterm clinical and radiographic outcomes. In younger children, early pin removal after adequate stability and radiographic union have been achieved appears to be a safe and feasible option that does not compromise clinical outcomes and may reduce the need for prolonged radiographic follow-up.

## 1. Introduction

Supracondylar humerus fractures are the most common elbow fractures in children, accounting for approximately 50% of all pediatric elbow fractures [1,2]. Furthermore, these fractures account for approximately two-thirds of hospital admissions for childhood fractures requiring surgical intervention [3].

The current treatment algorithm for these fractures is based on the Gartland classification system. The preferred treatment method for displaced supracondylar fractures is closed or open reduction followed by percutaneous pin fixation. Various authors have reported successful results with this method [4,5].

In the postoperative period, radiographic and clinical examinations play a fundamental role in assessing the maintenance of fracture reduction and whether the healing process is progressing appropriately. Following the achievement of sufficient clinical and radiological improvement, patients are gradually allowed to resume elbow joint motion after the removal of percutaneously placed Kirschner wires. However, there is no clear consensus in the literature regarding the timing of Kirschner wire removal. This decision is largely shaped by the surgeon’s clinical judgement and experience. Various studies have reported that the timing of Kirschner wire removal generally ranges between 3 and 6 weeks, with the widely accepted approach being removal in the 4th postoperative week [6–8].

The aim of this study was to compare the clinical and radiographic outcomes of pin removal performed between postoperative days 21–28 and those of pin removal performed after postoperative day 28 in pediatric patients treated with percutaneous Kirschner wires for supracondylar humerus fractures. It also aimed to contribute to the development of a standardized approach to the timing of pin removal.

## 2. Materials and methods

### 2.1. Study design

This retrospective study included a total of 90 pediatric patients aged 4–14 years who underwent surgical treatment for supracondylar humerus fractures at Ankara Etlik City Hospital between January 2023 and May 2025. All patients had Gartland types III and IV closed supracondylar humerus fractures. Patients were divided into two groups based on the timing of Kirschner wire removal: an early group, in which pin removal was performed between postoperative days 21 and 28, and a late group, in which pin removal was performed after postoperative day 28. The early pin removal group included 40 patients, while the late pin removal group included 50 patients.

To evaluate the effect of pin removal timing on treatment outcomes, clinical and radiographic outcomes were compared between the two groups. Additionally, to assess the possible effect of age, patients were divided into two age-based subgroups (≤8 years and >8 years), and early and late pin removal groups were compared within these subgroups. Patients with open fractures, flexion-type supracondylar humerus fractures, concomitant ipsilateral upper extremity fractures, previous surgery in the same region, incomplete clinical or radiographic data, or those whose parents could not provide written informed consent were excluded from the study. Demographic data, physical examination findings, and radiographic measurements were obtained retrospectively from hospital records.

### 2.2. Surgical technique

All patients were treated under general anesthesia with closed reduction and percutaneous Kirschner wire fixation. The diameter of the Kirschner wires used ranged from 1.5 to 2 mm. A standard cross-pinning configuration was applied in all patients. Reduction and pin placement were performed under fluoroscopic guidance.

### 2.3. Postoperative follow-up

All patients were monitored according to a standard follow-up protocol until the time of pin removal. Anteroposterior and lateral elbow radiographs were obtained within the first 5–7 days after surgery to assess the maintenance of reduction. To evaluate fracture healing, a second set of anteroposterior and lateral elbow radiographs was obtained between postoperative days 21–28. In the early pin removal group, Kirschner wires were removed following radiographic evaluation during this period. In patients who underwent delayed pin removal, a third set of anteroposterior and lateral elbow radiographs was obtained at the final outpatient visit, and pin removal was performed after radiographic evaluation. During follow-up, patients were evaluated in the early postoperative period immediately after pin removal and at 6 months postoperatively.

### 2.4. Radiological and clinical evaluation

Radiological evaluation was performed at 6 months postoperatively. In this evaluation, the lateral rotation percentage was measured to assess the quality of reduction and rotational alignment in the axial plane, whereas the Baumann angle was measured to assess alignment in the coronal plane. All measurements were performed independently by two pediatric orthopedic surgeons using standard anteroposterior and lateral elbow radiographs, and the mean values were used for analysis.

Clinical evaluation was performed in the early postoperative period immediately after pin removal and at 6 months postoperatively. To evaluate both functional and cosmetic outcomes, patients were assessed according to the Flynn criteria [9]. The results were compared with the healthy contralateral elbow using the Flynn criteria. The Mayo Elbow Score (MES) was also used for functional evaluation.

### 2.5. Ethical approval

This study was conducted in accordance with the ethical principles of the Declaration of Helsinki and its subsequent amendments. Ethical approval was obtained from the Ankara Etlik City Hospital Scientific Research Evaluation and Ethics Committee (approval no: AEŞH-BADEK1-2025-389, date: 27 August 2025). Written informed consent was obtained from the parents or legal guardians of all pediatric patients prior to inclusion in the study.

### 2.6. Statistical analysis

Statistical analyses were performed using IBM SPSS Statistics (version 26; IBM Corp., Armonk, NY, USA). The normality of the data distribution was assessed using the Kolmogorov–Smirnov test. The independent samples t-test and Fisher’s exact test were used for intergroup comparisons. The level of statistical significance was set at p < 0.05.

## 3. Results

The basic demographic characteristics and statistical analyses of the cases included in the study are summarized in Table 1.

The postoperative 6-month radiographic evaluation results and statistical analyses are summarized in Table 2.

All cases were divided into four groups according to the lateral rotation percentage (LRP):

Late group: LRP ≤ 10% (42, 84.0%), 10% < LRP ≤ 20% (7, 14.0%), 20% < LRP ≤ 30% (1, 2%), and LRP > 30% (0, 0%).

Early group: LRP ≤ 10% (26, 65.0%), 10% < LRP ≤ 20% (9, 22.5%), 20% < LRP ≤ 30% (3, 7.5%), and LRP > 30% (2, 5%).

The MES results and statistical analyses for patients in the early postoperative period and at 6 months are shown in Table 3.

The Flynn criteria results and comparative statistical analyses for patients in the early postoperative period and at 6 months postoperatively are presented in Table 4.

In age-based subgroup analyses of patients with early and late pin removal, no statistically significant differences were found between the groups in terms of Baumann angle and lateral rotation percentage measurements. In the clinical evaluation, a statistically significant difference was found between the groups in terms of the Mayo Elbow Score in the early postoperative period among patients aged ≤8 years (p = 0.01), whereas no significant difference was observed in patients older than 8 years (p = 0.72). In the 6-month evaluations, no statistically significant differences were found between the groups in terms of the Mayo Elbow Score in either age subgroup. In the age-based subgroup analyses performed using the Flynn criteria in the early postoperative period, a statistically significant difference was found between the groups among patients ≤8 years (p = 0.02), whereas no significant difference was observed in patients older than 8 years (p = 0.77). In the 6-month evaluations, no statistically significant differences were found between the groups in terms of the Flynn criteria in either age subgroup.

## 4. Discussion

There is no standardized approach in the literature regarding the timing of Kirschner wire removal after the surgical treatment of supracondylar humerus fractures, and practices vary among centers and surgeons. Some surgeons remove pins in the 2nd or 3rd week based on callus formation [10], whereas others remove them within 4 to 6 weeks [11]. In a 2019 study by Rupp et al., it was reported that pin removal could occur between 3 and 6 weeks [7]. In a 2022 survey conducted by Pavone et al. among members of the European Society for Pediatric Orthopedics, 58.2% of surgeons reported removing pins in the 4th week after surgery, whereas 40.2% reported removing them in the 3rd week [12]. This uncertainty in K-wire removal timing may lead to repeated clinical evaluations and serial radiographic examinations during follow-up, thereby increasing cumulative radiation exposure in pediatric patients. Furthermore, the high incidence and surgical management of this condition place an additional burden on the healthcare system. Additionally, prolonged retention of pins may delay functional recovery and increase the likelihood of pin-related complications. In this context, determining the effects of K-wire removal timing on clinical and radiographic outcomes is important for patient safety and efficient resource utilization.

In cases of delayed pin removal, prolonged rigid fixation may maintain angular alignment in the coronal plane; however, it may limit rotational control in the axial plane by reducing micromovements. Indeed, in supracondylar humerus fractures, coronal stability is largely achieved in the early period through pin configuration, and the Baumann angle shows minimal variability after healing begins; accordingly, no significant difference in Baumann angle was detected between the groups in the present study. In contrast, the lateral rotation percentage (LRP), which reflects the rotational alignment of the distal fragment in the axial plane, is a parameter that is more sensitive to the balance between fixation stability and physiological axial loading during healing. It was found to be significantly lower at the 6 month follow-up in patients who underwent early pin removal. In cases of prolonged pin retention, delayed physiological loading may have limited the development of rotational alignment in the axial plane, potentially contributing to greater rotational misalignment, even at a subclinical level. Although the difference in lateral rotation percentage between the early and late pin removal groups reached statistical significance, the absolute values in both groups remained relatively low. From a clinical perspective, such differences may not necessarily translate into meaningful functional impairment. Therefore, while the statistical difference is noteworthy, its clinical relevance should be interpreted cautiously.

Bone remodeling capacity is known to be significantly higher in children younger than 8 years compared with older age groups [13–15]. In our study, age-based subgroup analyses revealed significant differences in early postoperative clinical scores, particularly in patients aged ≤8 years, according to the Mayo Elbow Score and Flynn criteria. These findings suggest that the healing process in this age group may be more sensitive to the timing of pin removal.

The higher bone remodeling capacity in children aged ≤8 years [13,14] may explain why functional recovery is not adversely affected by earlier pin removal once adequate stability has been achieved, and may even support functional gains during the early stages of healing. Indeed, the absence of a significant difference in clinical outcomes between the early and late pin removal groups at 6 months suggests that early pin removal does not adversely affect midterm functional outcomes in these age groups and may be safely applied.

No statistically significant differences were found between the two groups in terms of the Mayo Elbow Score and the Flynn criteria in direct comparisons, either in the early postoperative period or at the 6 month follow-up. This suggests that both treatment approaches provide similarly satisfactory clinical outcomes in the overall patient population. The absence of significant differences between the early and late pin removal groups in terms of both radiological parameters and clinical outcomes suggests that earlier pin removal does not adversely affect functional or structural outcomes. This suggests that, in cases where adequate stability and radiographic union have been achieved, earlier pin removal may be a safe option that can be implemented without compromising clinical outcomes.

In the present study, radiation exposure was not directly measured. Therefore, the potential reduction in cumulative radiation exposure associated with earlier pin removal should be interpreted cautiously. However, earlier pin removal may reduce the need for additional follow-up radiographs in selected patients, potentially contributing to reduced radiation exposure in the pediatric population. In our study, the absence of statistically significant differences in both radiological and clinical outcomes between early and late pin removal suggests that, particularly in young children with high bone remodeling potential, pins may be removed earlier without compromising clinical outcomes, provided that adequate stability and radiographic union have been achieved. This approach has the potential to reduce the need for repeated radiographic examinations during follow-up. Particularly in young children, in whom hematopoietic activity is more widespread in long bones and sensitivity to ionizing radiation is higher, any attempt to reduce radiation dose should be considered clinically meaningful [16]. Indeed, the ALARA principle, adopted by the International Commission on Radiological Protection (ICRP) in 1977, defines the justification of radiation exposure and its maintenance at the lowest reasonably achievable dose levels as a fundamental principle [17].

This study has several methodological limitations. Its retrospective, single-center design inherently limited the standardization of certain variables. In addition, the timing of pin removal was determined according to the surgeon’s clinical judgment rather than a predefined protocol, which may have introduced selection bias between the groups. The timing of pin removal should therefore be considered when interpreting the results. The small number of patients in the age-based subgroup analyses and the follow-up period being limited to 6 months restrict the ability to draw conclusions about long-term outcomes. Furthermore, radiation exposure was not measured directly; the evaluation was based on indirect data derived from the number of radiographic examinations performed. Future studies with larger sample sizes and longer follow-up periods are needed to provide more robust data.

Ultimately, early pin removal may be considered a rational and potentially preferable treatment approach in the general pediatric population, with appropriate patient selection, as it may reduce cumulative radiation exposure and simplify the follow-up process.

## Figures and Tables

**Table 1:** Demographic characteristics.

Parameters	N (%)	p value
**Sex**		0.78
**Male**	47 (52.2)	
**Female**	43 (47.8)	
**Age**		0.66
**≤8**	51 (56.7)	
**>8**	39 (43.3)	
**Side**		0.68
**Right**	48 (53.3)	
**Left**	42 (46.7)	
**Dominance of the affected extremity**		0.82
**Dominant**	62 (68.9)	
**Nondominant**	28 (31.1)	

A p value of <0.05 was considered statistically significant.

**Table 2:** Radiological outcomes.

Parameters	Early group (N = 40) Mean ± SD	Late group (N = 50) Mean ± SD	p value
**Baumann angle**	71.6 ± 5.1	70.8 ± 5	0.62
**Lateral rotation percentage**	7.0 ± 5.0	11.1 ± 7.6	**0.03**

A p value of <0.05 was considered statistically significant.

**Table 3:** Mayo elbow score.

Mayo elbow score	Early group (N = 40) Mean ± SD	Late group (N = 50) Mean ± SD	p value
**Early postoperative period**	88.4 ± 6.2	84.7 ± 3.2	0.07
**6 month follow-up**	97.3 ± 1.9	96.3 ± 2.4	0.10

A p value of <0.05 was considered statistically significant.

**Table 4:** Flynn criteria.

	Early postoperative		6 month follow-up	
Flynn criteria	Early group N (%)	Late group N (%)	Early group N (%)	Late group N (%)
**Excellent**	30 (75)	38 (76)	34 (85)	40 (80)
**Good**	8 (20)	7 (14)	6 (15)	6 (12)
**Fair**	2 (5)	5 (10)	0(0)	4 (8)
**p value**	0.76		0.51	

A p value of <0.05 was considered statistically significant.
